# Modelling the effect of defects and cracks in solar cells’ performance using the d1MxP discrete model

**DOI:** 10.1038/s41598-023-39769-0

**Published:** 2023-08-01

**Authors:** Ricardo A. Marques Lameirinhas, Catarina P. Correia V. Bernardo, João Paulo N. Torres, Helena Isabel Veiga, Pedro Mendonça dos Santos

**Affiliations:** 1 Instituto Superior Técnico-Universidade de Lisboa, Av. Rovisco Pais, 1049-001 Lisbon, Portugal; 2 Instituto de Telecomunicações, Av. Rovisco Pais, Torre Norte, 1049-001 Lisbon, Portugal; 3 Academia Militar/CINAMIL, Av. Conde Castro Guimarães, Amadora, 2720-113 Lisbon, Portugal

**Keywords:** Solar energy, Photovoltaics, Solar cells

## Abstract

Renewable energies are increasingly playing an important role in the world’s energy supply. Society demands new solutions to solve environmental issues caused by fossil fuels. The importance of photovoltaic technology has been increasing and consequently, the necessity to have more accurate models to characterise the performance of solar cells during their entire lifetime has rose as well. Performance problems may appear during devices’ lifetimes associated with factors, such as weather conditions or faulty installation. Cracking might occur, leading to abrupt reductions on the produced power, quite difficult and expensive to fix. The I–V curves of a defected or cracked solar cell might not have the shape imposed by the usual models as 1M5P. In this article, cracked c-Si solar cells are modelled using a novel model: d1MxP. This model is based on the discretisation of the diode’s response on models as 1M5P. Instead of imposing a shape and compute some parameters to fit it on experimental data, the proposed model connects every two points. The results suggest a better fit using the proposed model in comparison with the 1M5P, not only in the original curves, but also modelling cracked cells. As consequence of a better fitting, the computation of important figures of merit as maximum power point or fill factor, reveals to be more precise. It is concluded that the proposed model might characterise the performance of a solar cell, even cracked, which is a huge advance aiming the possibility of simulating complex problems during the cells’ operation lifetime.

## Introduction

Fossil fuels have become the “punching bag” when analysing energy production and the future of our society on Earth. However, their importance on the human kind’s evolution is crucial. Firstly, the Industrial Revolution was triggered by the invention of stream engine, which is a mechanical device that produce movement by the combustion of coal or petroleum. It was an epic advance in the XVIII century. One century later, internal combustion engines were also invented, increasing the necessity from diesel and gasoline, composed by fossil fuels.

However, the combustion of fossil fuels and their derives had enormous negative impacts on the environment, leading to the climate crisis. As society’s needs and market demand lead to evolution, the focus on new energy sources has increased in the last decades. These new sources, associated with renewable energy, allow to overtake some disadvantages of energy production based on fossil fuels, namely the pollution caused on their combustion and the incapacity that the Earth has to regenerate that natural resource in comparison with the Human demand. Photovoltaic solar energy appears as one of the most impactful source, due to the relative low cost of production, operation and maintenance as well as the possibility of easy installation in entirely different locations on the planet^1–6^.

In 1839, Alexandre Edmond Becquerel demonstrated the photoelectric effect in a primordial device of a solar cell, which turned out to be contemporary with the developments of the industrial revolution and its technological discoveries. The successes and failures of these technologies are basically based on a single point: knowledge about them^1,4^. In that time, the knowledge about the physical phenomena behind the working principle of a solar cell was not sufficient to optimise a device capable to produce electrical power. In fact, the society has not had the necessity to have electricity. As example, the first public street lighting appeared in the early of XVIII century, based on coal and only in the final of that century some public buildings and streets had electrical based lighting systems. Of course, due to the society needs and its knowledge, fossil fuel combustion technologies had more significant advances than renewable technology ones, such as photovoltaic sources^1^.

The climate crisis creates a new society demand: the environmental impact of our actions. The analysis of a photovoltaic project has economic and financial indicators, but also environmental ones. That demand leads to massive advances, aiming the swift response to the challenge brought by the climate crisis. Solar cells are studied, from the fabrication processes, passing on the optimisation of their performance for different locations and applications, to their recycling stage^1,4–6^. Both simulation and experimental results are important to reach the goal of characterising this kind of optoelectronic device. Usually, simulation results allow us to understand the reality and to have a first insight of the system’s performance. Thus, the models’ accuracy is a goal when developing models to characterise the devices’ behaviour^1,4^.

Nowadays, different models to characterise the performance of a solar cell are used, such as 1M3P (1D3P), 1M5P (1D5P) or 1M7P (2D7P)^7–11^. Most of them are based on a current source in parallel with diodes and shunt and series resistances. Their accuracy has not been a problem. However, their accuracy is highly affected when modelling solar cells under extreme conditions (not standard/laboratory conditions) as defected/cracked solar cells, since the shapes of such characteristics do not follow the standard shapes of those models^1,4,5^.

In this article, a new model is proposed based on the discretization of the diode electrical response, named as d1MxP. This model gives us a model rigorous characterisation of the solar cell’s performance, since it connects every two adjacent points of an I–V curve, instead of obtaining a certain number of parameters based on specific I–V points to fit results in a certain shape. For that reason, the obtained results suggested a better fitting to experimental results, not only on original cells but also after progressive crack formation. Cells’ outputs and figures of merit are computed better, as the maximum power point, the fill factor, efficiency and conversion yields. Thus, the proposed model is capable to characterise the performance of a solar cell, even if it is cracked, which is a huge advance aiming the possibility of simulate complex problems during the cells’ operation lifetime in a given photovoltaic system.

## Methodology

**Figure 1:**
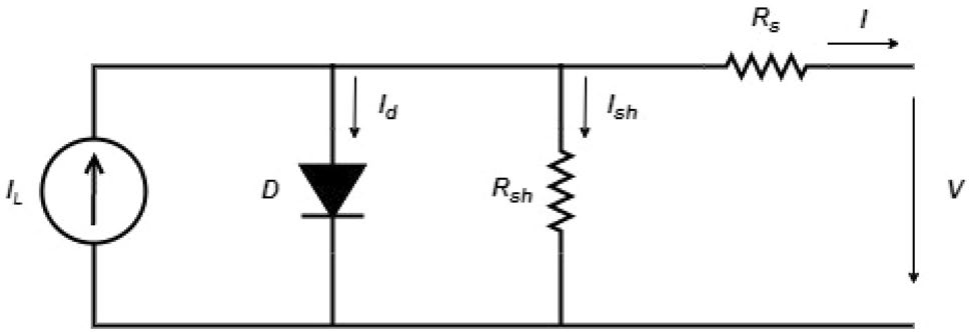
1M5P model’s equivalent circuit.

The d1MxP model used consists of the discretisation of an electrical model used to represent the behaviour of solar devices. In this case, the 1M5P model is used as the basis, whose equivalent circuit is shown in Fig. 1, because it is a simple model to analyse, it is widely used and it takes into account the internal losses of the system^1–3^.

Applying Kirchhoff’s laws, the output current *I*, as a function of the output voltage *V* is given by Eq. (1), being $I_L$ the photogenerated current, $I_d$ the diode’s current, $I_o$ the diode reverse current (also known as inverse saturation current or dark current), *n* the diode non-ideality factor, $R_s$ the series resistance associated to a voltage loss due to the cell’s connections and $R_{sh}$ the shunt/parallel resistance related to a current loss due to the current leaks on the cell^1–3,7,8^.

1 $$\begin{aligned} I = I_L - I_d -I_{sh}= I_L - I_o \left( e^{\frac{V+R_sI}{nV_T}}-1\right) - \frac{V+R_sI}{R_{sh}} \end{aligned}$$

The 1M5P model is characterised by five parameters ($R_s$, $R_{sh}$, $I_o$, *n* and $I_L$), being obtained through equations already presented in the literature^1,8^. Each of them is obtained through a restricted range of points, which translates into a disadvantage of this model, given that the ability to portray the behaviour of photovoltaic devices ends up being affected. Other similar models are in the literature^2,3,9–11^. However, this approach is general to all of them, since it is based on the diode discretisation.

In contrast, the d1MxP model takes into account the total set of discrete experimental points, considering the performance between each two adjacent points, which, from an electrical point of view, translates into the decomposition of the diode into its own equivalent model, consisting of an ideal diode in series with a resistance $R_\gamma$, and an independent ideal voltage source $V_\gamma$.

The ideal diode in each branch, when conducting, collects current from the current source, decreasing the cell’s output current. The branch is active (ideal diode conducts) when the output voltage is higher than the voltage $V_\gamma$ of the branch source, being the slope of the branch of the I–V curve proportional to $-\,1/R_\gamma$^2,3^.

**Figure 2:**
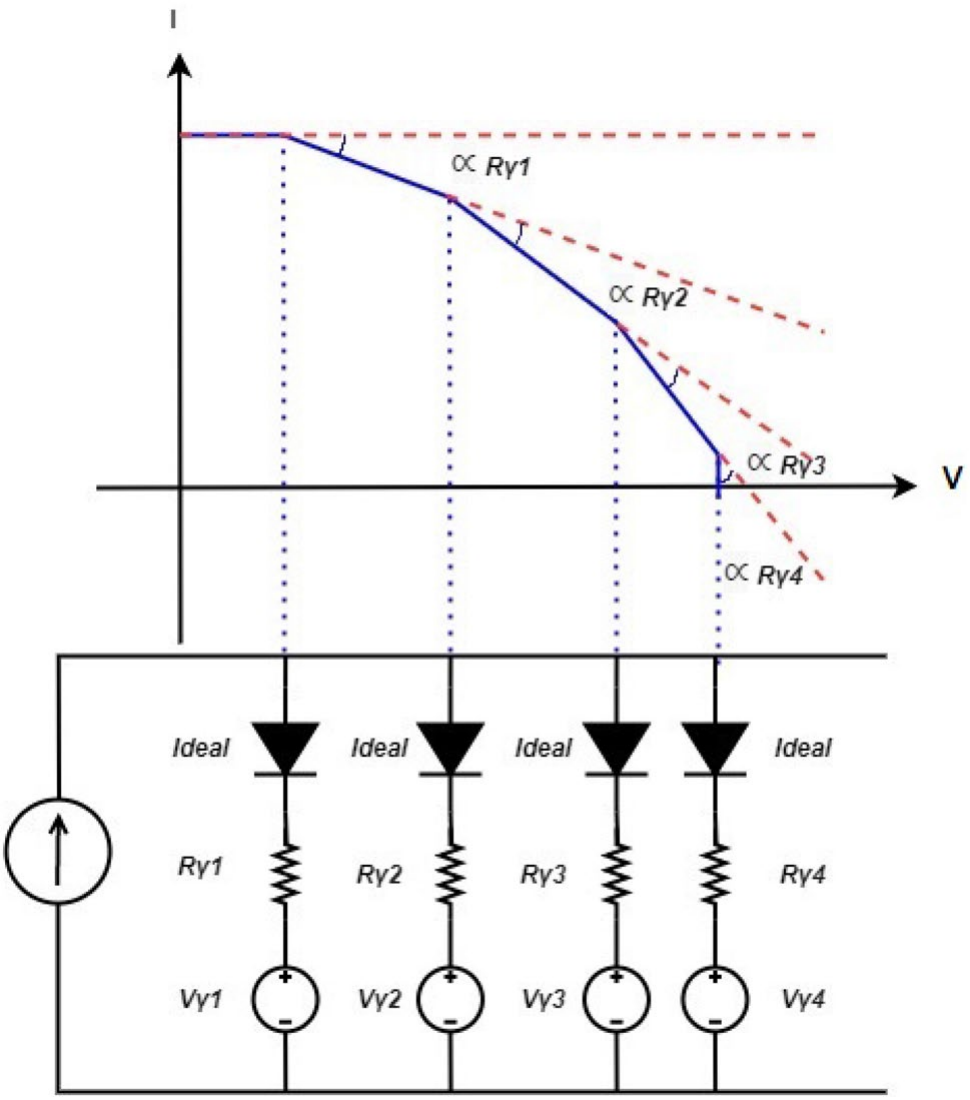
I–V curve of the d1MxP model: N branches in parallel (example for N = 4).

Thus, it is possible to connect every two points of the cell characteristic curve, as illustrated in Fig. 2 followed by the equivalent circuit of the model with N branches, with the slope between the points depending not only on the resistance of the N-th branch, but also on the previously activated (N−1)-th parallel branches. Mathematically, it can be described by the equations set 2, where *m* is the slope between each two points^2,3^.

2 $$\begin{aligned} {\left\{ \begin{array}{ll} V_\gamma = V_i\\ R_\gamma = -\frac{1+m_im_{i-1}}{m_i-m_{i-1}} \end{array}\right. } \end{aligned}$$

It is important to note that the d1MxP model assumes that the slope between every two points on the I–V curve decreases as the output resistive load increases, so the model excludes the experimental points where this does not happen and, once there might be a number of significant experimental points, the model results should follow the behaviour of the experimental characteristic curves, minimising the associated error^2,3^.

## Results

The ageing effect of cells and their degradation and defects, including possible cracks in the semiconductor connections and in the cells themselves, has several repercussions on the proper performance of solar devices^4^. The developed model is applied to several c-Si experimental tests, already published^4^. The c-Si solar cells are from mono crystalline silicon, known by the AK50X50 model of Aiyima. Firstly, it was applied to the intact c-Si cells (original I–V curve), without any type of crack and, then with progressive crack degradation, the ”crack1” example being the one that presents the lowest cracking scenario and the ”crack5” situation the one that presents a higher percentage of cracking. The experimental procedure and data analysis is already presented^4^. The aim of this article is not to go as deep as the analysis performed before^4^, but it is to present the model as a novel approach to characterise a complex effect, taking conclusions about the modelling process.

**Figure 3:**
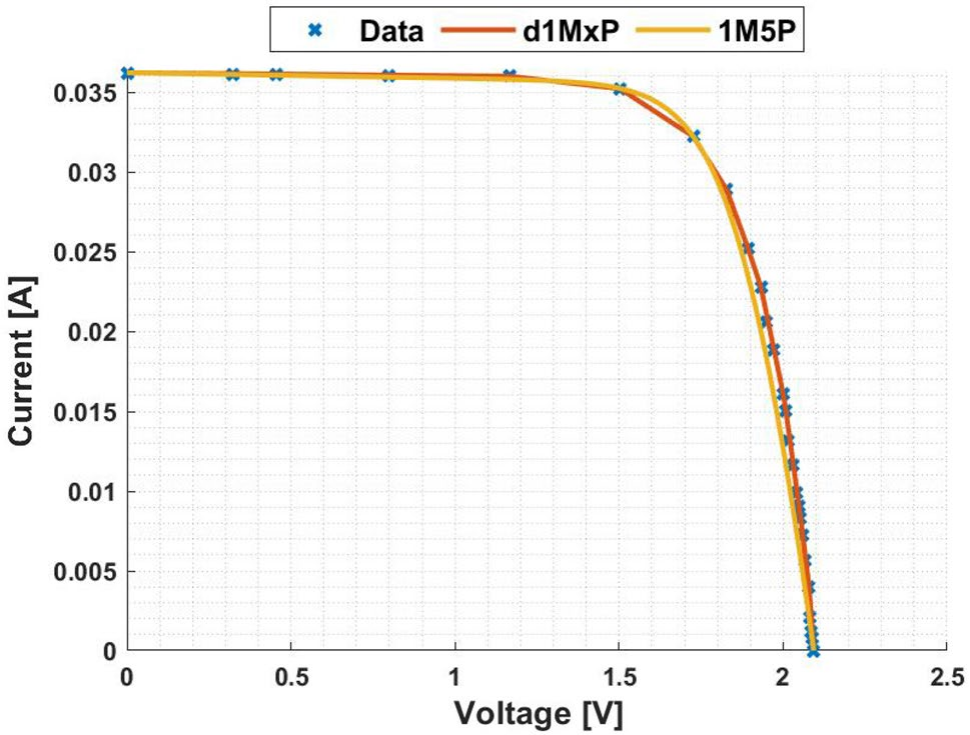
I–V curve at original situation.

**Figure 4:**
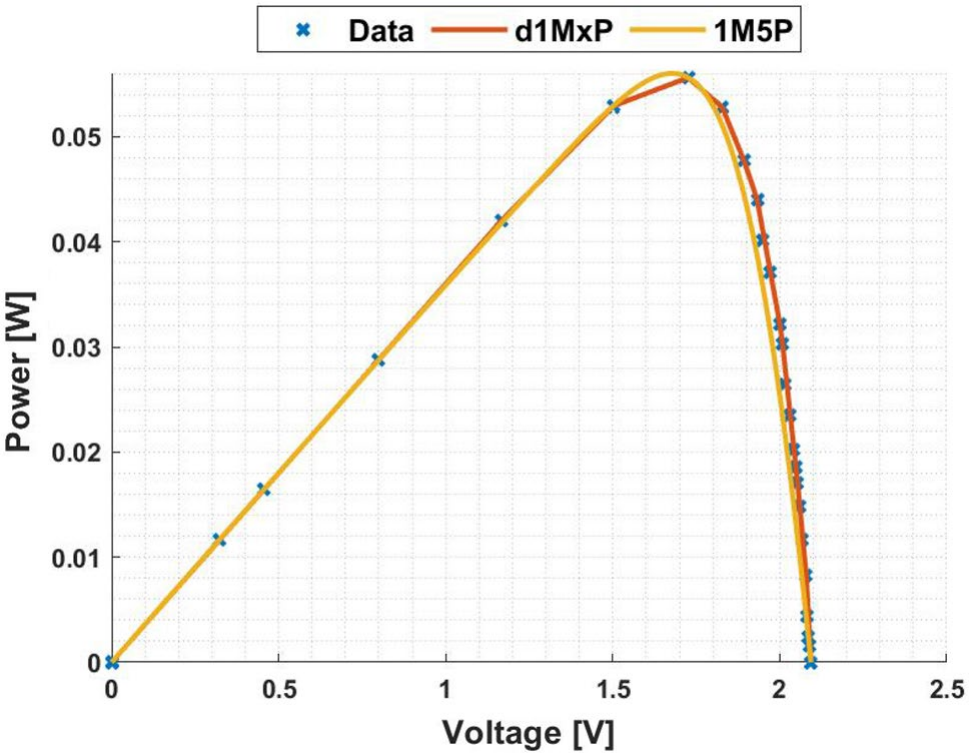
P–V curve at original situation.

In Figs. 3 and 4 are presented the c-Si characteristics curves of the solar cells without cracking effect, using the proposed model, d1MxP, and the 1M5P model, as well as the experimental points. For the original case, i.e. the case in which the set of cells is intact, the five parameters of 1M5P model are: $I_L =$ 0.0363 A, $I_o =$1.0797 $\times$ 10$^{-10}$ A, $n=$1.0382, $R_{sh}=$ 2.8983 $\times$ 10$^{3}$ $\Omega$ and $R_s =$ 3.6320 $\Omega$.

With the experimental data, the discrete model has 11 branches and 22 parameters, which means that the proposed model can be called d1M22P. The parameters’ values are presented in table 1.

**Table 1:** Branches connected in parallel for original situation.

N	$R_{\gamma }$ [$\Omega$]	$V_{\gamma }$ [V]
1	5237.2881	0.0000
2	471.0790	1.1667
3	89.8556	1.5028
4	51.7432	1.7260
5	42.3490	1.8277
6	186.7045	1.8927
7	27.5458	1.9322
8	38.6027	2.0000
9	40.1011	2.0085
10	56.0208	2.0537
11	9.1147	2.0791

Observing the characteristic curves it can be seen that, in this situation, both models fit the experimental points. However, because they are experimental points they do not follow the linearity of the 1M5P model. On the other hand, the discrete model follows the experimental data, reducing the associated error.

**Figure 5:**
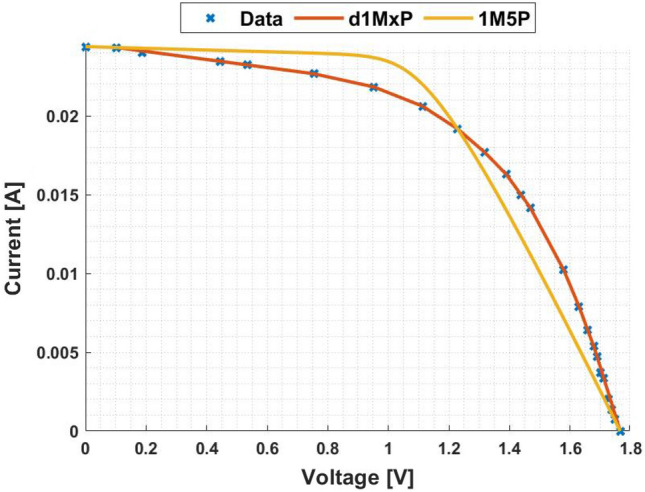
I–V curve at “crack1” situation.

In Fig. 5, it is represented the 24 points obtained from^4^ at “crack1” situation. In this case, the five parameters of 1M5P model are computed as $I_L =$ 0.0247 A, $I_o =$8.5813 $\times$ 10$^{-17}$ A, $n=$0.5164, $R_{sh}=$ 1.8305 $\times$ 10$^{3}$ $\Omega$ and $R_s =$ 23.4681 $\Omega$, which I–V curve is also presented in this figure. It is also presented in Fig. 5, in red, the I–V curve computed using the d1MxP model.

As can be seen in the I–V curve, the d1MxP model approximates the experimental points obtained, bringing the characteristic curve closer to the real behaviour of the solar cell. Thus, there is a significant reduction in the error when using the discrete model compared to the 1M5P one. For the lowest values of voltage, the discrete model is able to follow the experimental data, in contrast to the 1M5P model, which has an associated error. Graphically, the error is described by an abrupt current drop.

**Figure 6:**
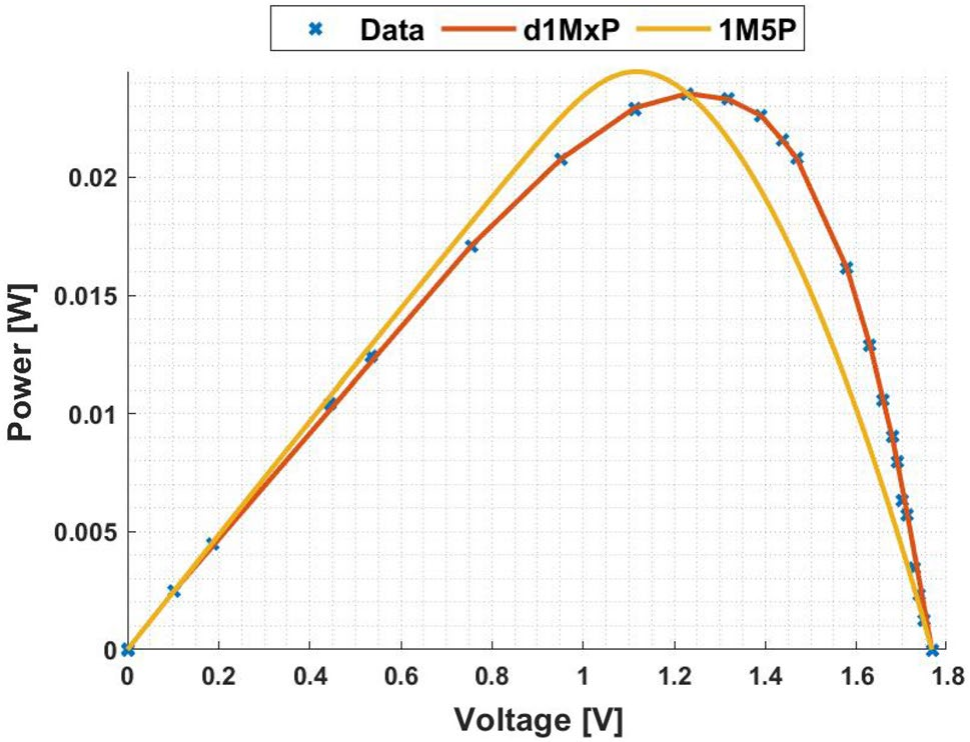
P–V curve at “crack1” situation.

Based on the experimental data, the d1MxP model has 15 branches and 30 parameters (x = 30), whose values are presented in Table 2. Since the reduction of the associated error in the I–V curve is verified, the same is observed in the P–V curve, presented in Fig. 6.

**Table 2:** Branches connected in parallel for “crack1” situation.

N	$R_{\gamma }$ [$\Omega$]	$V_{\gamma }$ [V]
1	1830.4576	0
2	498.0580	0.1017
3	602.3533	0.7542
4	296.1866	0.9520
5	204.8996	1.1130
6	242.6388	1.2288
7	325.5299	1.3192
8	144.1432	1.3898
9	4757.6298	1.4379
10	111.4005	1.4689
11	99.3154	1.5791
12	162.4635	1.6299
13	144.5103	1.6780
14	408.2547	1.6893
15	1551.6627	1.7119

**Figure 7:**
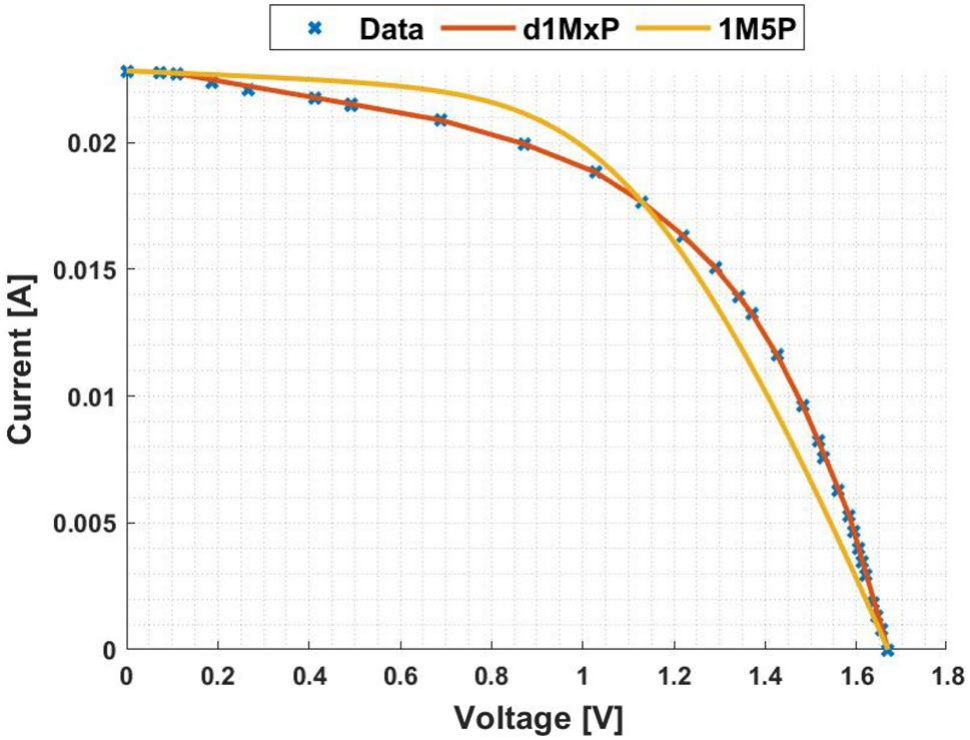
I–V curve at “crack2” situation.

**Figure 8:**
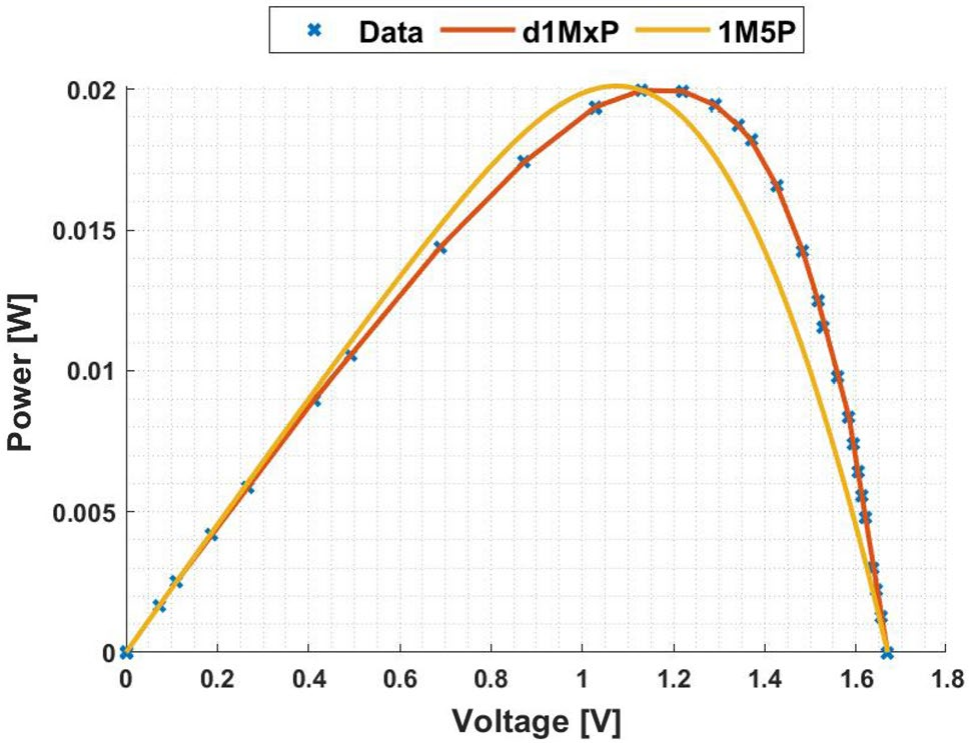
P–V curve at “crack2” situation.

For the “crack2” situation, the I–V and P–V characteristic curves of the solar cell are shown in Figs. 7 and 8, respectively, and, in this crack situation, the five parameters of the 1M5P model are $I_L =$ 0.0231 A, $I_o =$1.0224 $\times$ 10$^{-7}$ A, $n=$1.3230, $R_{sh}=$ 1.3220 $\times$ 10$^{3}$ $\Omega$ and $R_s =$ 18.1598 $\Omega$. It is possible to verify that the curves presented corroborate the statements of the analysis carried out previously. The parameters of the N branches of the discrete model are presented in Table 3. The model has 32 parameters and it can be called d1M32P.

**Table 3:** Branches connected in parallel for “crack2” situation.

N	$R_{\gamma }$ [$\Omega$]	$V_{\gamma }$ [V]
1	1321.9831	0
2	1322.0863	0.0734
3	620.4311	0.1102
4	11591.1259	0.4124
5	517.4180	0.6893
6	498.0448	0.8729
7	231.4626	1.0282
8	305.1364	1.1299
9	299.1825	1.2203
10	266.1675	1.2910
11	572.3289	1.3418
12	169.6095	1.3701
13	169.6685	1.4266
14	179.7219	1.4831
15	407.5035	1.5169
16	53.2017	1.5847

**Figure 9:**
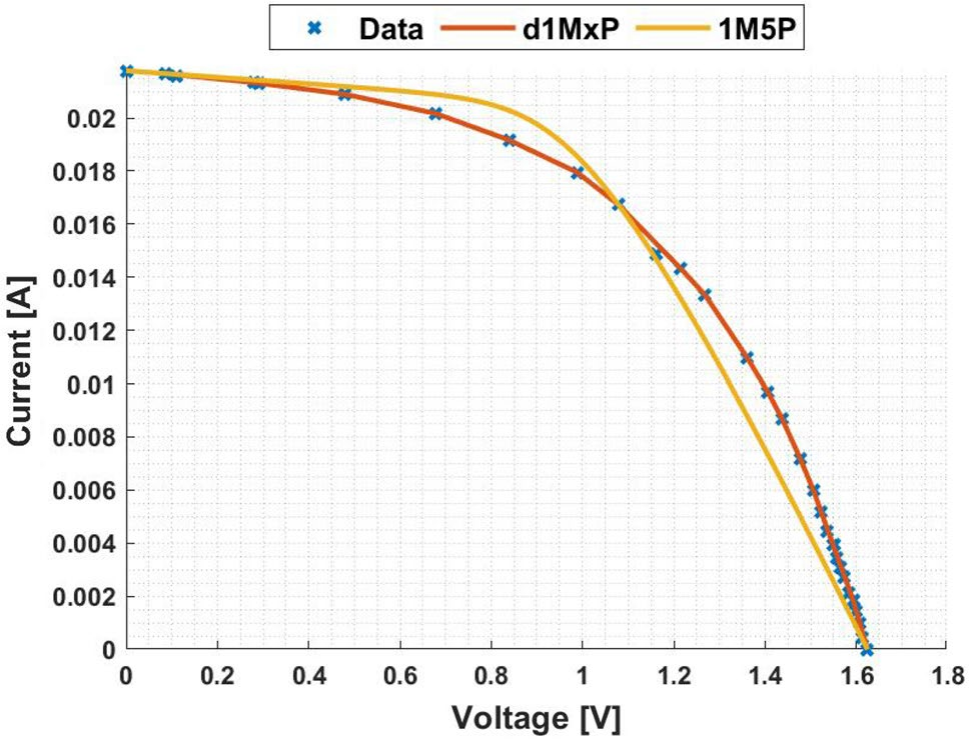
I–V curve at “crack3” situation.

**Figure 10:**
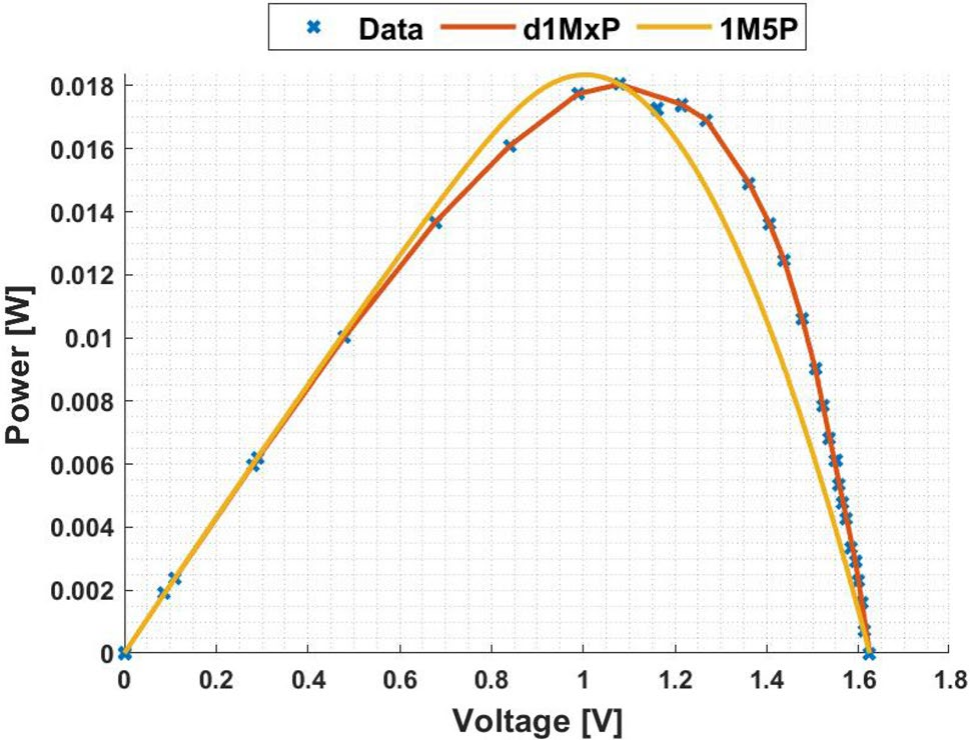
P–V curve at “crack3” situation.

Figures 9 and 10 present the I–V and P–V curves of the solar cell, respectively, in the ”crack3” scenario as well as the d1MxP. The 1M5P parameters are computed as $I_L =$ 0.0225 A, $I_o =$3.2067 $\times$ 10$^{-12}$ A, $n=$0.6999, $R_{sh}=$ 788.1356 $\Omega$ and $R_s =$ 25.4237 $\Omega$. The parameters of the discrete model used are in Table 4, and, in this situation, x, which corresponds to the number of parameters, is 34. Once again, the use of the discrete model translates into a reduction of the associated error, which is an advantage of this model over others.

**Table 4:** Branches connected in parallel for “crack3” situation.

N	$R_{\gamma }$ [$\Omega$]	$V_{\gamma }$ [V]
1	788.1102	0
2	2143.9217	0.0876
3	2080.4447	0.2797
4	696.1816	0.4802
5	390.9171	0.6780
6	512.0599	0.8390
7	186.6814	0.9887
8	244.1259	1.0791
9	986.9809	1.2147
10	143.0340	1.2684
11	378.4072	1.3616
12	255.9227	1.4068
13	174.2237	1.4379
14	297.0748	1.4774
15	159.5322	1.5056
16	87.8165	1.5932
17	3.3632 $\times$ 10$^{15}$	1.6073

**Figure 11:**
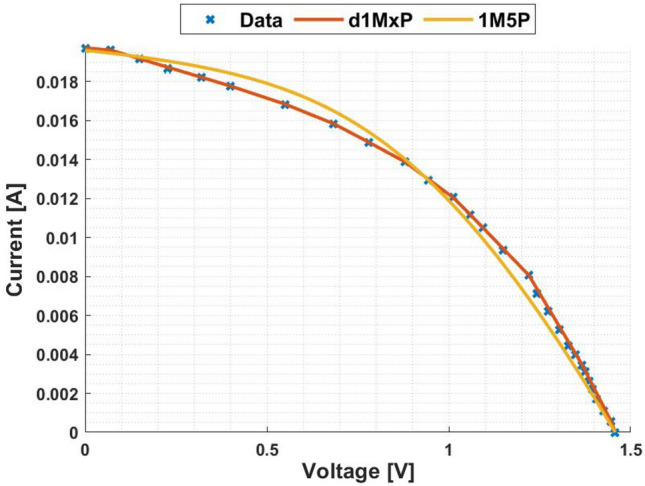
I–V curve at Crack 4 situation.

**Figure 12:**
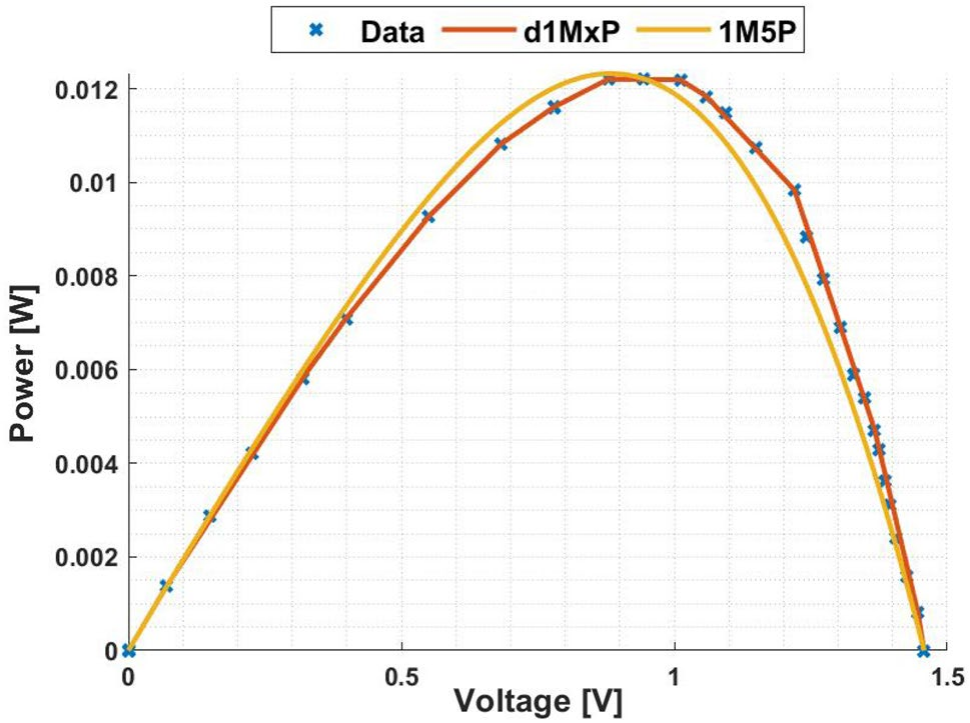
P–V curve at ”crack4” situation.

At “crack4” situation, the 1M5P parameters are $I_L =$ 0.0203 A, $I_o =$1.8237 $\times$ 10$^{-5}$ A, $n=$2.0554, $R_{sh}=$ 635.5932 $\Omega$ and $R_s =$ 20.3390 $\Omega$, and the I–V curve of the solar cell is shown in Fig. 11 as well as the experimental data’s I–V curve and the one resulting from the d1MxP model, being $\times$ equal to 26. The parameters of the N branches of the d1MxP model are presented in Table 5. The power characteristic curves are presented in Fig. 12, and these curves corroborate, once again, the statements of the previous analysis.

**Table 5:** Branches connected in parallel for “crack4” situation.

N	$R_{\gamma }$ [$\Omega$]	$V_{\gamma }$ [V]
1	635.5678	0
2	249.1473	0.0706
3	31323.0172	0.3192
4	1747.3643	0.3983
5	745.9934	0.5508
6	434.5468	0.6836
7	3559.6760	0.7797
8	270.7306	0.8785
9	212.7618	1.0113
10	1232.2202	1.0593
11	82.9045	1.2203
12	195.0494	1.3672
13	79.2381	1.4463

**Figure 13:**
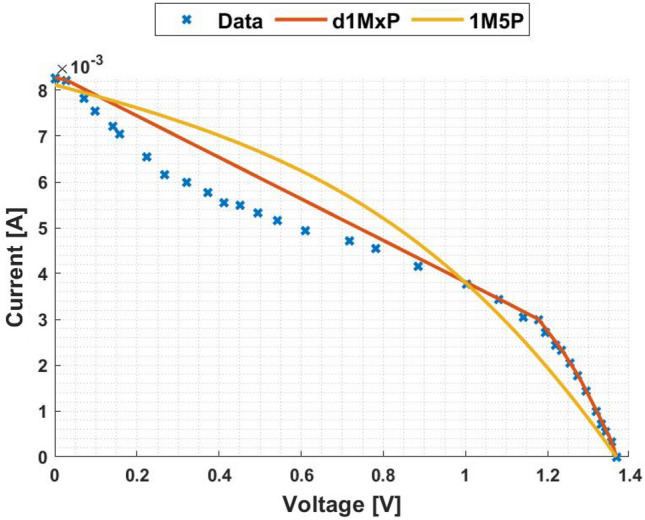
I–V curve at “crack5” situation.

**Figure 14:**
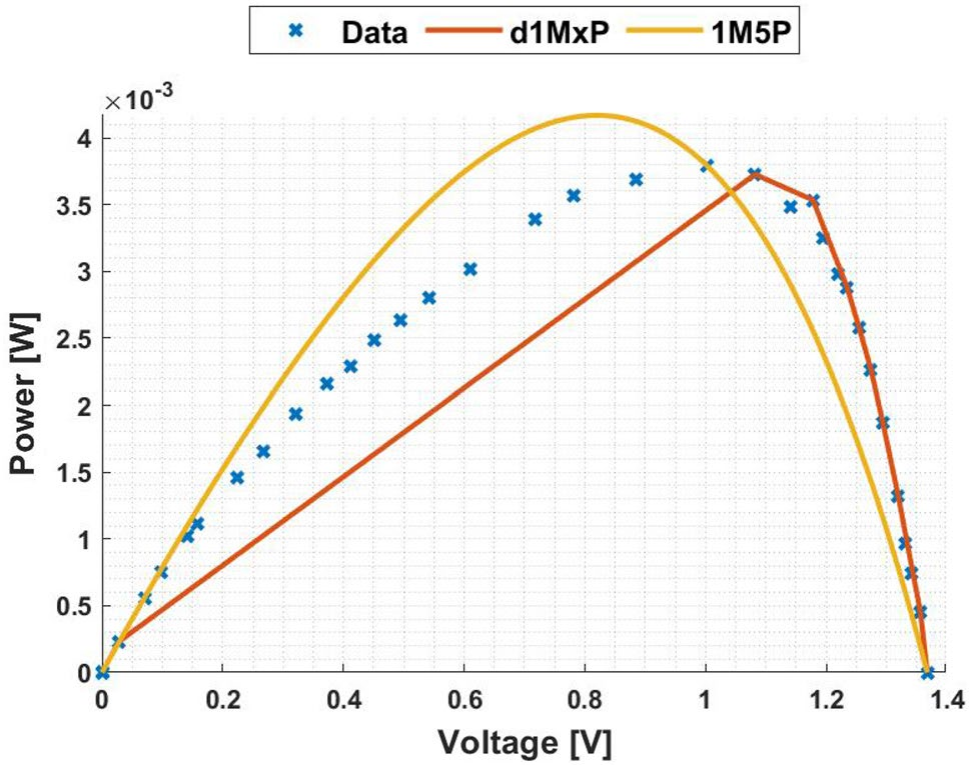
P–V curve at “crack5” situation.

Lastly, the I–V curves of both models, including the experimental data, for the “crack5” situation are presented in Fig. 13. In this situation, the formation of this crack leads to the disconnection of parts of the cell and when these areas are completely disconnected from the cell’s electrical circuit, these ones become inactive. As far as the experimental points are concerned, the slope between every two points does not become successively more negative, so the model cannot pass through most of the experimental data. However, the fact that parts of the cell are disconnected from each other and the low current range in which the device can operate, leads to the existence of systematic errors that justify the increase in errors associated with the d1MxP model. The 1M5P parameters are computed as $I_L =$ 0.0088 A, $I_o =$6.9187 $\times$ 10$^{-5}$ A, $n=$2.9646, $R_{sh}=$ 508.4746 $\Omega$ and $R_s =$ 33.8983 $\Omega$, and for the d1MxP the 20 parameters are presented in Table 6. The P-V curves can be observed in Fig. 14 and it can be seen that the discrete model continues to be more precise than the 1M5P model, which is an advantage on the maximum power point determination.

**Table 6:** Branches connected in parallel for “crack5” situation.

N	$R_{\gamma }$ [$\Omega$]	$V_{\gamma }$ [V]
1	508.4695	0
2	389.4541	0.0282
3	10747.6831	1.0819
4	139.4281	1.1780
5	444.9890	1.2345
6	4.4352$\times$10$^{16}$	1.2542
7	356.0165	1.2740
8	1602.1669	1.2938
9	1487.7601	1.3192
10	88.1828	1.3559

For the analyses carried out, it can be seen that increasing the percentage of cracking in the cells causes not only a decrease in the short-circuit current, but also a decrease in the open-circuit voltage. Consequently, the 1M5P model’s resistances suffer alterations. The shunt resistance value, $R_{sh}$, decreases due to the generation of high conductivity parallel paths that cross the p-n junction or the corners of the cell, and the series resistance value, $R_s$, increases. The only situation where this is not verified is the “crack2” situation, due to $R_s$ having a higher value than the one in “crack1”. However, this is a result of experimental errors associated with the measurement.

In Table 7 the maximum values of power, voltage and current for the original situation and for the five cracks situations under analysis, using both models, are shown.

**Table 7:** The maximum power ($P_{max}$), maximum voltage ($V_{max}$) and maximum current ($I_{max}$).

		d1MxP	1M5P
Original	$P_{max}$ [W]	0.0556	0.0560
	$V_{max}$ [V]	1.7260	1.6775
	$I_{max}$ [A]	0.0322	0.0334
Crack 1	$P_{max}$ [W]	0.0236	0.0245
	$V_{max}$ [V]	1.2288	1.1173
	$I_{max}$ [A]	0.0192	0.0219
Crack 2	$P_{max}$ [W]	0.0200	0.0201
	$V_{max}$ [V]	1.1299	1.0748
	$I_{max}$ [A]	0.0177	0.0187
Crack 3	$P_{max}$ [W]	0.0180	0.0183
	$V_{max}$ [V]	1.0791	1.0040
	$I_{max}$ [A]	0.0167	0.0183
Crack 4	$P_{max}$ [W]	0.0122	0.0123
	$V_{max}$ [V]	0.8785	0.8839
	$I_{max}$ [A]	0.0139	0.0139
Crack 5	$P_{max}$ [W]	0.0037	0.0042
	$V_{max}$ [V]	1.0819	0.8198
	$I_{max}$ [A]	0.0034	0.0051

Regarding the Fill Factor, the values obtained for the d1MxP model are presented in Table 8, as well as the value of the experimental Fill Factor recorded in the literature^4^. The discrete model leads to an increase in the Fill Factor values since this model is more accurate. It is important to know that the literature’s Fill Factor is determined using the 1M3P formula^4^, instead of the 1M5P one, meaning that the curve is treated as a rectangle. Thus, this factor constitutes an advantage of the discrete model, since the Fill Factor is computed using the trapezoids below the used points.

**Table 8:** Fill factors.

	Data (ref.^4^)	d1MxP
Original	0.7323	0.9180
Crack 1	0.5542	0.7980
Crack 2	0.5294	0.7820
Crack 3	0.5093	0.7757
Crack 4	0.4307	0.6976
Crack 5	0.3629	0.6194

**Figure 15:**
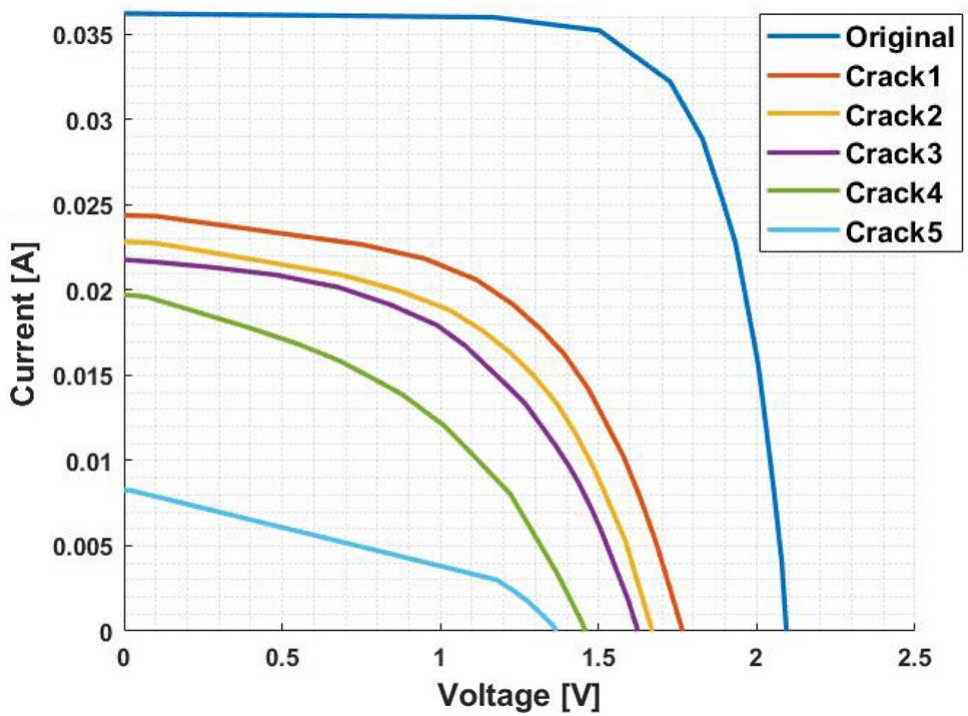
I–V curves of the d1MxP model.

**Figure 16:**
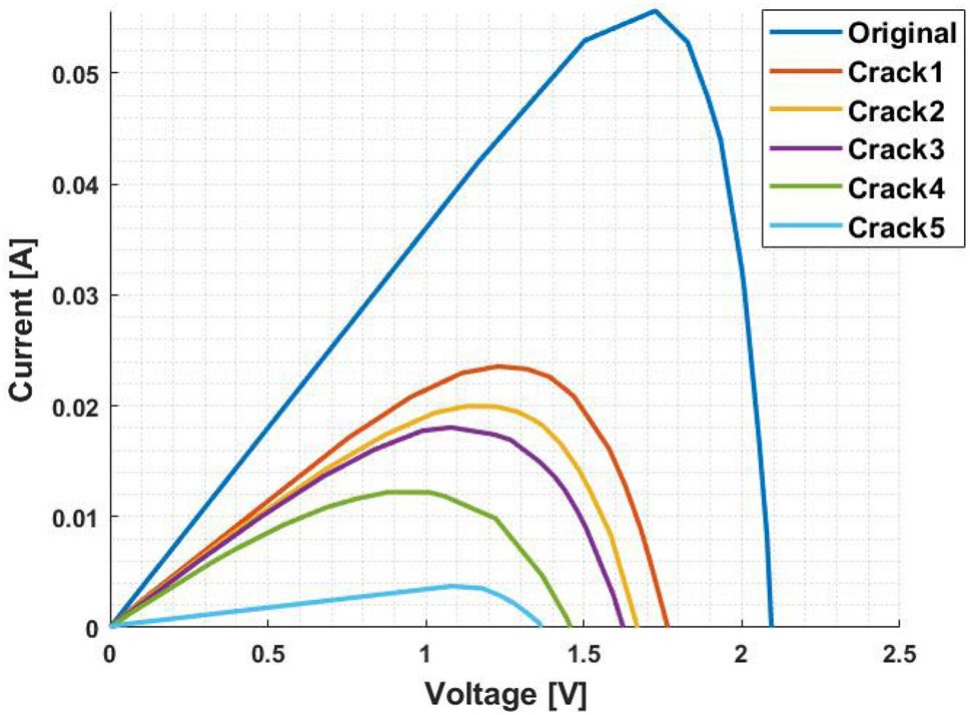
P–V curves of the d1MxP model.

For all five crack situations and for the original one, Figs. 15 and 16 show the behaviour of the d1MxP model. It is verified that the model tracks the significant impact that degradation and crack situations have on the good behaviour of solar devices. Increasing the percentage of cracking leads to a change in the shape of the characteristic curves, given that, as occurs in the “crack5” situation, the crack ends up being responsible for the disconnection of parts of the cell, and electrically, it ends up translating into a lower linearity of the points.

## Conclusion

The cracking effect is a factor responsible for decreasing the performance of solar devices, decreasing the current produced by itself due to the possibility of disconnection of parts of the cell from its electrical circuit. Because of this, there is a need to have models that best fit the performance of the cells, minimising the associated error.

The discrete model d1MxP consists of the discretisation of the 1M5P model, being possible to fit the characteristic curves to the experimental data, decreasing the associated error, in particular at the maximum power point and the Fill Factor, if compared to traditional models like 1M5P.

With the analysis carried out, it can be seen that, even in situations where the device behaviour is not so linear and/or ideal, the proposed model is more accurate and precise in relation to the experimental data, since it corresponds to a micro-scale analysis, taking into account each set of adjacent points, which contrasts with the 1M5P model whose characteristic curves are determined taking into account macro parameters. This demonstrates the model’s ability to adjust to different operating conditions with adverse effects on its performance.

## Data Availability

The datasets used and/or analysed during the current study available from the corresponding author on reasonable request.
